# Supplementary material for: Whole genome sequences of multi-drug resistant Escherichia coli isolated in a Pastoralist Community of Western Uganda: Phylogenomic changes, virulence and resistant genes
Source: PLoS One. 2020 May 29;15(5):e0231852. doi: 10.1371/journal.pone.0231852 (PMC7259651; doi:10.1371/journal.pone.0231852)
Supplement: Attachment — Submitted filename: Response to Reviewers.docx [file pone.0231852.s001.docx]

**Response to Reviewers**

**Editor’s comments**

| **SN** | **COMMENT** | **AUTHOR’S RESPONSE** |
| --- | --- | --- |
| 1 | Please ensure that your manuscript meets PLOS ONE's style requirements, including those for file naming. | This has been corrected as recommended |
| 2 | Please provide additional details regarding participant consent. In the ethics statement in the Methods and online submission information, please ensure that you have specified how verbal consent was documented and witnessed. | Additional details regarding participant consent and the ethical statement |
| 3 | We note that you have stated that you will provide repository information for your data at acceptance. Should your manuscript be accepted for publication, we will hold it until you provide the relevant accession numbers or DOIs necessary to access your data. If you wish to make changes to your Data Availability statement, please describe these changes in your cover letter and we will update your Data Availability statement to reflect the information you provide. | The data availability statement has been updated. |
| 4 | Your ethics statement must appear in the Methods section of your manuscript. If your ethics statement is written in any section besides the Methods, please move it to the Methods section and delete it from any other section. Please also ensure that your ethics statement is included in your manuscript, as the ethics section of your online submission will not be published alongside your manuscript | The ethical statement has been relocated to the methods |
| 5 | Please ensure that you refer to Figure 5 in your text as, if accepted, production will need this reference to link the reader to the figure | Figure 5 has been referenced in the text |
| 6 | Please either i) remove the figure or ii) supply a replacement figure that complies with the CC BY 4.0 license. | The figure has been removed |

**Reviewer 1**

| **SN** | **COMMENT** | **AUTHOR’S RESPONSE** |
| --- | --- | --- |
| **1** | Please highlight the exact number of tested strains in abstract. Cattle (n=12) and human (n=30). In abstract we can found: […] 21/42 isolates were ESBL producers; 13/42 from human and 8/42 from cattle – it remains unclear, and suggest there were 84 samples. I suggest to use 13/30 and 8/12 instead, respectively. | The exact numbers tested have been highlighted as recommended by the reviewer. Thank you |
| **2** | Please indicate the exact number of tested strains in Methods section, subsection Bacterial strains as mentioned above | Amended as recommended by the reviewer |
| **3** | Major conclusion made by Authors stays a little bit contrary to data provided in Figure 2. According to Figure 2 about 8 cattle E. coli strains (60% of all cattle samples) are closely related to this found in human and the several of them have similar or wider pattern of AMR. | The conclusion has been amended as to reflect the results. |
| **4** | Could you explain why only cattle was designated to the study? If the cattle is the most important meat donor in Uganda, such thing should be mentioned in the background section. | The pastoralists in Kasese district majorly reared cattle. This has been mention in the manuscript |
| **5** | Consider discuss other work i.e work of Okubo et al (1) in meaning possibility of obtaining AMR genes from other livestock | This has been used to enrich the discussion, thank you. |
| **6** | Because of relative small number of samples in case of cattle (n=12), and no comparison to other livestock (lack of discussion i.e abovementioned) the conclusion may be misleading. | This has acknowledged as a limitation |
| **7** | consider the necessity of Figure 1. It is not essential for study. | The figure has been deleted |
| **8** | Method section: Whole genome-sequencing: “Briefly, gDNA was fragmented and tagged with […]” | The reference has been added |

**Reviewer 2**

| **SN** | **COMMENT** | **AUTHOR’S RESPONSE** |
| --- | --- | --- |
| **1** | In Manuscript draft there are missing keywords, however there are present in main document so I assumed that this is omission. In some places like in page 5 Escherichia coli is not written in italic. When you once use shortcut "E. coli" in the text do not use full name in further part. You should write "ng/µl" not "ng/ul" (page 4). | Key words have been added and all other comments addressed as recommended by the reviewer |
| **2** | In Results I thought that it will be more genetic data, knowing the title of article. That part could be improved. In addition despite the fact that the Discussion is written correctly and in scientific way, it also could be improved. For a research article the Discussion is to short, try write more about importance of your results regarding to other papers. | Some adjustments have been made |
